# The numerosity and mean size of multiple objects are perceived independently and in parallel

**DOI:** 10.1371/journal.pone.0185452

**Published:** 2017-09-28

**Authors:** Igor S. Utochkin, Konstantin O. Vostrikov

**Affiliations:** National Research University Higher School of Economics, Moscow, Russia; University of Naples Federico II, ITALY

## Abstract

It is well documented that people are good at the rapid representation of multiple objects in the form of ensemble summary statistics of different types (numerosity, the average feature, the variance of features, etc.). However, there is not enough clarity regarding the links between statistical domains. The relations between different-type summaries (numerosity and the mean) are of particular interest, since they can shed light on (1) a very general functional organization of ensemble processing and (2) mechanisms of statistical computations (whether averaging takes into account numerical information, as in regular statistics). Here, we show no correlation between the precision of estimated numerosity and that of the estimated mean. We also found that people are very good at dividing attention between numerosity and the mean size of a single set (Experiment 1); however, they show some cost of dividing attention between two same-type (two numerosities or two mean sizes, Experiment 2) and two different-type (one numerosity and one mean size, Experiment 3) summaries when each summary is ascribed to a different set. These results support the idea of domain specificity of numerosity and mean size perception, which also implies that, unlike regular statistics, computing the mean does not require numerosity information. We also conclude that computational capacity of ensemble statistics is more limited by encoding several ensembles than computing several summaries.

## Introduction

Numerous studies have shown that the visual system is very efficient at rapid representation of multiple objects in the form of *ensemble summary statistics* such as numerosity [1–5], the mean along a dimension–size ([6,7], etc.), orientation [8–10], color [11,12], speed [13], brightness [14], even emotional expression and gender [15,16], as well as variance [17–19]. There is evidence that the summary statistics are encoded directly as visual properties rather than inferred by any means, as they are prone to adaptation aftereffects [1,18,20]. The ensemble summaries are computable when conscious access to individual objects is limited [6,10,21,22]. Neurophysiological data also support the notion that the representation of ensembles as summaries is not based on the properties of individual objects [23,24].

Ensemble summary statistics give us some evident advantages. One such advantage is that they serve as better estimates of the set in general than each single item does [25]. Another advantage is that the summaries are efficient means for organizing multiple items into chunks [26–29] and for surviving the severe limitations of the processing bottleneck. Although it is a subject of debate whether extracting summary statistics is based on a genuine parallel representation of all presented items at one time [6,25,30–36] or on limited capacity sampling of just a few objects [37–41], it is generally admitted that it leads to fairly efficient approximation of multiple properties in the entire display in terms of both precision [6,25,32,42,43] and speed [7,34,44].

### The functional structure of ensemble statistics: Correlation and parallelism

Many of the previously reported studies tested the properties and mechanisms of ensemble perception within separate domains (e.g., average size, average orientation, and numerosity). Together, they showed robustness in core phenomenology–an ability to judge generalized statistical features at a brief glance with relatively good accuracy. This finding naturally leads to the question of whether these local domains are linked together. One possibility is that there is high processing specialization between domains (for example, retinotopic feature maps, which are supposed to be rather independent in the visual cortex, or *modular* [45]), so computing statistical summaries is a part of processing within each module. Alternatively, it is possible that there is a locus within the cognitive system where different domains overlap, and some shared factors affect the resulting ensemble perception.

One approach to probing the functional relations between the domains is measuring *correlations* between tasks across observers [46,47]. The logic of measuring correlations is straightforward: if the tasks are correlated, then the domains addressed by these tasks have some overlap; if they are not correlated, they are likely separate and not supported by any common mechanism. In their recent study, Haberman, Brady, and Alvarez [48] implemented this approach in the field of ensemble perception. Specifically, they were interested in whether there are any common mechanisms/factors underlying ensemble averaging across a variety of visual domains. The authors tested the precision of reporting the average color; average orientation in two types of objects (Gabor patches and isosceles triangles); average emotional expression and average identity of human faces. In each experiment, Haberman et al. [48] ran a pair of tasks using the same participants. In total, eight experiments allowed the estimation of pairwise correlations between the majority of the tested summaries. Haberman et al. [48] found high correlations between the ‘low-level’ summaries (color and orientations), as well as between the ‘high-level’ summaries (emotional expression and face identity). However, cross-level correlations were low: The precision of color or orientation averaging did not correlate with that of emotion or face identity. Moreover, these cross-level correlations did not exceed a correlation between a summary statistics task and a verbal working memory task requiring no ensemble representation. This result led Haberman et al. [48] to conclude that there is no single domain-general averaging mechanism that could analyze information from all visual modules. At the same time, their results show some narrower generality spreading within the levels of the visual hierarchy (for example, for computing mean color and mean orientation). However, the authors admit that the conclusion drawn from the latter result is ambiguous. On one hand, this result can indicate the existence of a common computational statistical mechanism within each level. On the other hand, the correlations can be explained by some shared source of noise arising between features lying closer in the representational space (e.g., color and orientation) but not between remote features (e.g., orientation and facial expression).

Apart from testing correlations between tasks, there is another useful approach that is relevant for studying the functional organization. We will refer to it as testing *parallelism*. While correlational studies concern the overlap between different domains, parallelism studies raise a rather different question. Whether two domains overlap or not, it is important to know how they are coordinated in gaining access to conscious perception. For example, if we look at cars on a road trying to estimate their mean speed and mean size at one time, how would the information from the two domains be processed? Will we observe the same efficiency as reported in the numerous ensemble studies within separate domains–unlimited capacity, or parallel processing? Or, will we observe decrement in judging one or both summaries, suggesting interference between the domains? In terms of the functional organization, parallelism addresses the question whether a *limited-capacity bottleneck* is present anywhere in the system and makes various domains compete to be fully implemented.

Studies on task parallelism for probing functional organization have a long history in the field of attention [49–52]. As the role of attention in ensemble perception is a subject of ongoing discussion [8,21,25,53], a number of studies have also addressed the existence of the limited-capacity bottleneck in processing several summaries. Some of these studies have focused on an ability to process more than one summary along a single dimension (for example, the mean sizes of two color sets of dots). Chong and Treisman [54] have shown that it is possible to estimate the average sizes of two ensembles in parallel as accurately as the average size of only one ensemble: participants in their experiment were equally good at judging the average size of a relevant color set when the color was both precued (attention is paid to a relevant set) and postcued (attention is divided between the sets). Later, other studies have shown that no more than two [28] mean sizes can be stored in working memory and that probably only one mean size or mean orientation can be perfectly attended to at one time [30,31]. Halberda et al. [5] have also shown the two-set limit for parallel processing of several numerosities (plus superset numerosity that is always available, regardless of the number of sets). Note that, based on these results, it is difficult to say whether observed limitations in parallel computation reflect the bottleneck in multiple statistical computations or in the division of attention between different sets. From the object perception literature, it is known that it is harder to attend to different objects than to different features of one object [55].

More important for the issue of functional organization are the studies where division of attention between two different summaries are tested. Emmanouil and Treisman [56] showed that dividing attention between the mean size and the mean speed or between the mean size and the mean orientation yields a cost in performance that is not found in the division of attention between two mean sizes. Moreover, the cost is even more dramatic if the mean size is to be estimated in one set and the mean speed (or orientation) is to be estimated in another set. Huang [57] showed that a cost of dividing attention between the orientation summary (prevalence of vertical items over horizontal ones or vice versa) of one ensemble and the color summary (prevalence of reddish items over greenish ones or vice versa) of another is comparable with the cost of dividing attention between features of different objects. An idea that the observed limits of parallel statistical processing has more to do with limits in encoding several sets than statistics per se is supported by other researchers [58,59]. This concept complicates conclusions about the single vs. multiple processors. We will address these complications in our experiments.

### Numerosity and the mean

In previous section, we reviewed the studies probing the functional links between ensemble summaries of the same type, mostly two means (or prevalence [57], which can be considered as just an alternative measure of the central tendency). However, for a more comprehensive view of the architecture underlying ensemble summary statistics, it is necessary to address the link between summaries of different types, e.g., numerosity and the mean.

Although Haberman et al. [48] demonstrated that even two summaries of the same type can be computed independently (e.g., mean orientation and mean facial expression), it does not necessarily imply that two summaries of different types are also independent. This doubt comes from regular mathematical statistics. You can measure two or more attributes in a sample and calculate mean values from each of them, so that each such calculation will be independent from others (e.g., you do not need to know the mean size to calculate the mean speed). However, you cannot calculate the mean without knowing the number of observations (numerosity). Similarly, you cannot calculate the variance without knowing the mean and the number. Therefore, according to regular statistics, summaries of the same type can be calculated independently (which is also the case in ensemble perception [48]), but summaries of different types cannot. Is that also true for ensemble summary statistics? It is easy to see that answering this question can not only shed light not only on between-domain links, it can also inform us about the nature of computations in the core of statistical representations in vision (although we admit that this is an indirect way to study these computations).

Recently, Lee, Baek, and Chong [60] addressed the link between these different types of summaries. Their participants had to estimate the average size, the number, or the total area of a set of circles. The authors then estimated how well each of the parameters could be predicted by the remaining two parameters. They found that the total area is predicted by both the mean size and numerosity much better than other summaries are predicted by any of the rest. This finding led the authors to conclude that the mean size and numerosity can be processed independently, and the total area is computed as the mean multiplied by numerosity.

The study by Lee et al. [60] is an example of the correlational approach to the functional organization of ensemble perception. However, the authors tested the perception of numerosity, mean, and total area in separate blocks, so their paradigm required participants to pay attention to one summary at a time. In our opinion, however, parallelism should also be tested for a more complete view of the visual statistical system at work. Given the independence between numerosity and the mean size obtained in the separate tests [60], we can ask how these two systems are coordinated when attempting to extract different summaries from the same stimulus. Is there a processing bottleneck limiting their concurrent efficiency? Or, in contrast, can they work in parallel, providing lossless computation of both numerosity and the mean? We will address this issue in our experiments.

### Our study

As noted above, Lee and colleagues [60] made the first step to shape the functional relationships between the mean and numerosity. Their findings are based on the correlational design. Here, we combine the correlational approach with that aimed at testing parallelism. It is based on the precue-postcue paradigm. Earlier, Emmanouil and Treisman [56] and Huang [57] used this paradigm to test whether two same-type summaries (two means or two prevalence statistics in different sensory domains) can be estimated together without loss in performance. We use this paradigm to test coordination between two different-type summaries. Combining both tests–correlation and parallelism–in one study has some important advantages. First, it allows deeper probing of the functional structure of ensemble summary statistics, because two different aspects of that structure are tested rather than one. Second, measuring each summary under two different conditions–precue and postcue–provides additional inputs for evaluating in the correlational matrix. Instead of looking at one correlation between two separately measured rates (as in typical correlational studies, e.g., [46, 48, 60]), we can look at precue-postcue correlations within each summary and compare two between-summary correlations observed under different cue conditions. The precue-postcue correlations are informative because they can show whether our tests are reliable and whether the tested abilities are robust across tasks. In turn, comparing the between-summary correlations can show whether the whole tested structure is reliable and stable. In other words, it can show whether an attempt to process two summaries at once changes the functional links between these two domains.

Based on this approach, we conducted three experiments testing correlation and parallelism in the perception of numerosity and mean size. In Experiment 1, we tested how these two summaries are computed within a single set. Experiments 2 and 3 aimed to compare the efficiency of concurrent computation of two of the same summaries (mean-mean or numerosity-numerosity) with the efficiency of concurrent computation of two different summaries (mean-numerosity). Since it is impossible to assign two mean sizes or two numerosities to the same set, in both Experiment 2 and 3, we used two sets with only one relevant parameter assigned to each set. Together with Experiment 1, this allowed dissociation of the cost caused by attending to two summaries from that caused by attending to two sets.

## Experiment 1

### Method

#### Participants

For determining the sample size, we used the statistical tool G*Power 3.0.10 [61]. We set required statistical power at .8, a Type I error at .05, and an expected Cohen’s *d* at .6. The expected effect size was set based on the previous research of cue effects on numerosity and mean size perception [5,56,59]. These values led us to a minimum sample size of nineteen participants. Considering a possibility of technical problems or poor performance in some participants, we recruited twenty-three participants. Psychology students of the Higher School of Economics (HSE) took part in the experiment for extra course credit (14 females, ages ranging between 18 and 22 years). All participants reported having normal or corrected-to-normal vision and no neurological problems. The participants were unaware of the purpose of the experiment. Before beginning the experiment, they signed an informed consent form for the procedure, which had been approved by the Institutional Review Board at HSE.

#### Apparatus and stimuli

Stimulation was developed and presented through PsychoPy [62] for Linux. Stimuli were presented on a standard VGA monitor at a refresh frequency of 75 Hz with 800 × 600-pixel spatial resolution. A homogeneous gray field subtending approximately 19.44° × 19.44° was used for stimulus presentation. The rest of the screen space was black and not used for presentation. The “working” gray field was divided into 6 × 6 imaginary cells, each having a side length of 3.24°. These imaginary cells served for item positioning, as described in the next paragraph.

Filled white circles with different diameters were used as items in a sample set. Each circle could be located within one of the 36 imaginary cells, and each cell could be occupied by only one circle or left empty. Each circle could be placed at a random position within a cell with a restriction that its center be located at least one radius + .16° away from any cell border along cardinal axes. This restriction meant that no circle ever overlapped with another. On the other hand, sets looked rather random in terms of spatial arrangement.

The number of circles could vary between 7 and 36; random cells were chosen on each trial for placing the circles. The diameters of the circles were randomly drawn from the following list: .86°, 1.03°, 1.19°, 1.35°, 1.51°, 1.67°, and 1.84°. The mean diameter of the circles in each drawn display ranged between trials from 1.08° to 1.67°. The standard deviation of the diameter in each display ranged from .14° to .21°.

For precue, the Russian equivalents of either the word “NUMBER” or “MEAN” were used. The words were printed in white Arial (letter height 1.6°) and located in the center of the screen. For postcue, six question marks were presented instead of the words. For numerosity reports, a white cursor appeared below the gray “working” field to type in a number. For mean size reports, a white adjustable probe circle was presented at the center of the screen. The starting size of the probe varied randomly between .43° and 2.21°.

#### Procedure

Experimental sessions were run in a darkened room. Participants were seated approximately 50 cm from a monitor. They were instructed to estimate and report the number or/and the mean size of a set of briefly presented circles.

**Fig 1:**
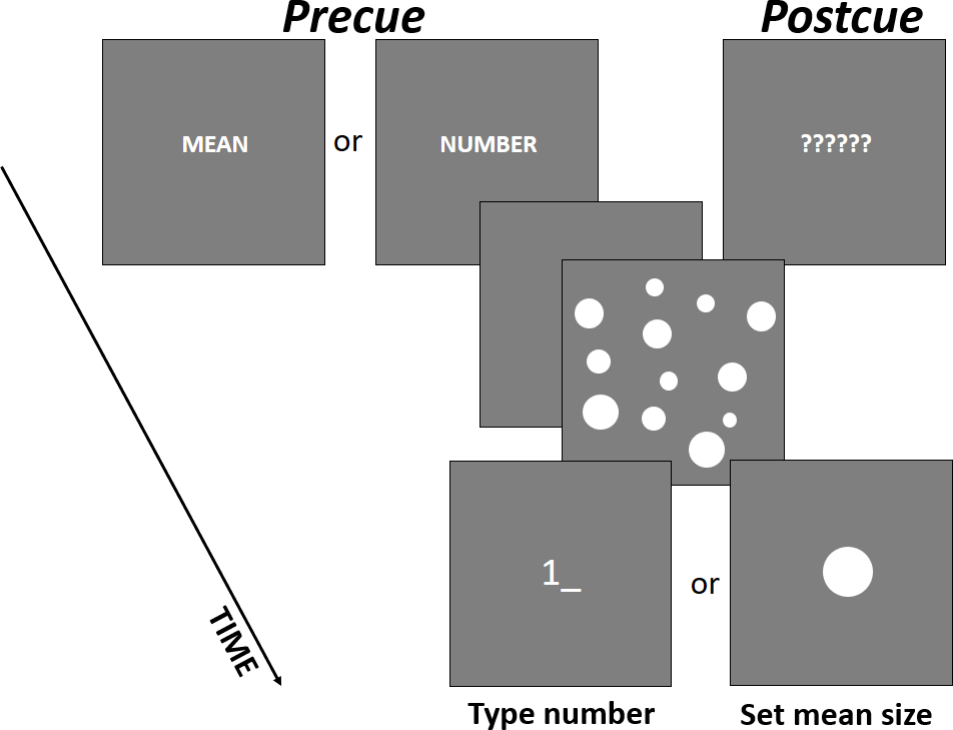
An example of a single trial with time course in Experiment 1. (A) precue and (B) postcue conditions.

Each trial started with a presentation of a cue screen for 500 ms that also served for gaze fixation on the center of a screen. In the precue trials, a word, either “NUMBER” or “AVERAGE,” appeared saying which of the summaries were to be reported in the end of the trial (Fig 1). In the postcue trials, six question marks appeared warning that the relevant parameter would be known after sample presentation (Fig 1). The cue screen was followed by a 500-ms blank screen that was, in turn, followed by the presentation of a sample set for another 500 ms. Immediately after the sample, a test screen was presented. The participants had to type in the number of circles in the sample [5] or adjust a probe circle to match the average size of the circles in the sample [38]. The appearance of the probe screen (a cursor for typing or the probe circle) also informed the participants about their task in the postcue trials (Fig 1). For typing the numbers, a numerical pad of a standard keyboard was used (arrow, “DEL”, and “BACKSPACE” keys could be used for editing an entry). To increase or decrease the size of the probe pixel by pixel, right and left arrow keys were used. The time for response was not limited. To confirm their response and quit the trial, participants had to press ENTER. The next trial started upon pressing SPACE, so participants could progress at a comfortable pace and take a rest whenever they wanted.

#### Design and data analysis

In this experiment, we used a 2 (Cue–precue vs. postcue) × 2 (Task–numerosity vs. mean size estimation) within-subject design. The number of trials per condition was 60 (two repetitions of 30 displays covering the whole range of numbers from 7 to 36). An experimental block, therefore, consisted of 2 × 2 × 60 = 240 trials presented in a random order. The experimental block was preceded by 8 practice trials that were not included in the data analysis.

For the analysis, a relative error magnitude was calculated on each trial as an absolute deviation of the observer’s response from the correct response divided by the correct response. The relative error was considered to characterize the precision of numerosity or mean size judgments.

Two types of analysis were then applied to the relative errors. First, we estimated an effect of Cue on each of the tasks using a standard t-test along with a Bayesian t-test. The latter is considered to be an alternative to classical significance testing [63] and is based on comparing evidence for H_1_ against H_0_. The H_1_-to-H_0_ likelihood ratio–the Bayes factor (*BF*_10_)–is a principal quantitative measure of evidence of one hypothesis over another [64,65]. Bayesian analysis was run using JASP software [66]. The prior effect size distribution was set as a Cauchy with a median of 0 and a width of .707, a default setting recommended when no information about the expected effect size is available in advance [66]. To interpret the Bayes factors, we used Jeffreys’ scale [64] with Kass and Raftery’s adjustment [65], where 1 < BF_10_ < 3 is evidence for H_1_ that is “not worth more than a bare mention” ([65], p. 777), where 3 < BF_10_ < 20 is positive evidence for H_1_, 20 < BF_10_ < 150 is strong evidence for H_1_, and where BF_10_ > 150 is very strong evidence for H1; the inverse ratios define symmetrical evidence ranges for H_0_.

Second, a correlation analysis was applied to the data. Averaged relative error values were also calculated for each participant for all four factorial cells. We then estimated correlations between these four data points across participants. There were four correlations of particular interest for us. Correlations between the same type of summaries in different cue conditions (“Numerosity—Precue” with “Numerosity—Postcue”, and “Mean size—Precue” with “Mean size—Postcue”) were to estimate consistency of the tested abilities across cue conditions. Correlations between different summaries at same-cue conditions (“Numerosity—Precue” with “Mean size—Precue”, and “Numerosity—Postcue” with “Mean size—Postcue”) were to show if any interrelation exists between numerosity and mean size estimation. Again, two methods were applied for judging whether the correlations exist: (1) the standard significance tests and (2) computing Bayes factors for estimating whether posterior distributions of correlation coefficients more likely have a higher (H_0_) or lower (H_1_) density around 0 than a homogeneous prior distribution (beta-distribution with a width of 1 [JASP Team]).

### Results and discussion

**Fig 2:**
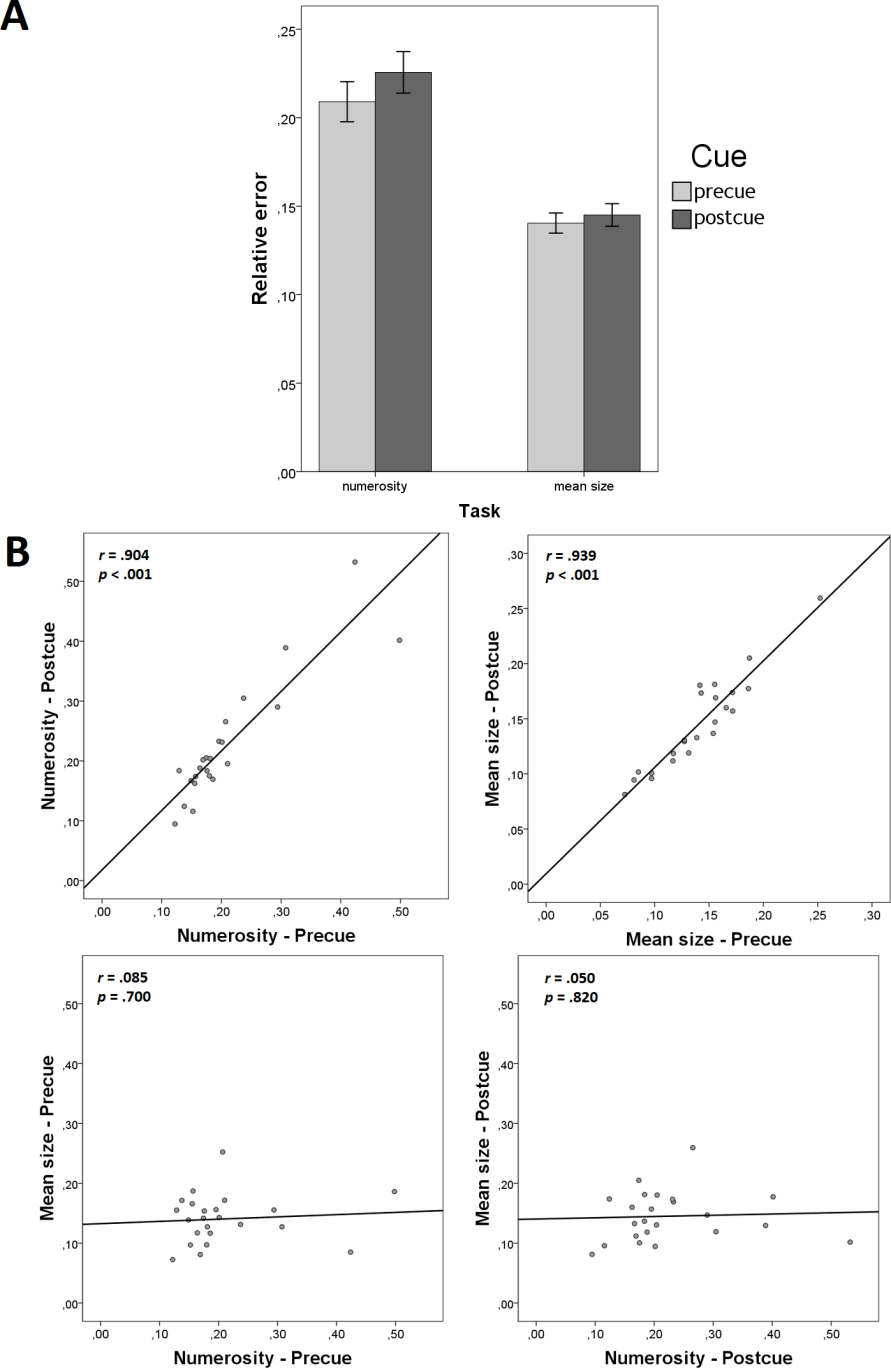
Results of Experiment 1. (A) cue effects on the relative error of numerosity and mean size judgments (error bars denote ±95% CI); and scatterplots showing (B) precue-postcue correlations within summaries and (C) between-summary correlations within different cue conditions.

We found no effects of Cue on the relative error (Fig 2A), both in the numerosity task (*t*(22) = 1.486, *p* = .151, *d* = .310, *BF*_10_ = .573) and in the mean size task (*t*(22) = 1.540, *p* = .138, *d* = .321, *BF*_10_ = .613). Therefore, Experiment 1 showed no evidence for cost of dividing attention between the two summaries.

Fig 2B and 2C illustrate the informative correlations. We found very high positive precue-postcue correlations within each summary (“Numerosity—Precue” with “Numerosity—Postcue”: *r* = .904, *p* < .001, *BF*_10_ = 3.349 × 10^6^; “Mean size—Precue” with “Mean size—Postcue”: *r* = .939, *p* < .001, *BF*_10_ = 1.566 × 10^8^; Fig 2B). These correlations show high consistency between the two conditions in which each summary is measured. From a more general viewpoint, they suggest that the individual differences in both measured abilities–enumeration and averaging–are robust against cue manipulations. In contrast, we found no correlations between different summaries within same-cue conditions (“Numerosity—Precue” with “Mean size—Precue”: *r* = .085, *p* = .700, *BF*_10_ = .277; “Numerosity—Postcue” with “Mean size—Postcue”: *r* = .050, *p* = .820, *BF*_10_ = .265; Fig 2C). This lack of correlation suggests the absence of a common mechanism for the perception of numerosity and the mean size.

In sum, two results of Experiment 1 are most important in terms of the topic. First, we found no cost of division of attention between the numerosity and the mean size of a set. Second, we found no correlation between the precision of judging numerosity and the mean. Taken together, these findings suggest that these two tasks can be carried out in parallel and independently of each other, at least when both summaries are estimated within one set of objects. This is consistent with the idea of non-overlapping well-coordinated mechanisms for computing numerosity and the mean size.

In Experiment 1, observers had to compute two summaries at one time (in the postcue trials). Adopting the idea of the separate computational mechanisms, we assume that each computation is carried independently and share no processing bottleneck. This allows to avoid interference. We can call it *distributed computation*. In the next experiments, we compare this distributed computation (two concurrent computations, each is performed by a separate mechanism) with a situation where a single processor is busy computing two summaries (that we can call *shared computation*). We suppose that shared computation occurs when two same-type summaries are estimated. In Experiment 2, we tested this in a task requiring the distribution of attention between two numerosities or two mean sizes. Obviously, it is impossible to have two same-type summaries within one set, so two sets are always required. As we noted earlier, this causes certain problems in interpretation, because it is hard to dissociate between parallel statistical computations and parallel attending to two sets [56,57]. For this reason, it would be difficult to compare Experiment 2 (same summary in two sets) with Experiment 1 (different summaries in one set). In Experiment 3, we solved this problem by introducing combined conditions, namely, different summaries in different sets.

## Experiment 2

### Method

#### Participants

Nineteen psychology students of the Higher School of Economics took part in the experiment for extra course credit (10 females, ages ranging between 18 and 22 years). All reported having normal or corrected-to-normal vision and no neurological problems. The participants were unaware of the purpose of the experiment. Before beginning the experiment, they signed an informed consent for the procedure approved by the Institutional Review Board at HSE.

#### Apparatus and stimuli

**Fig 3:**
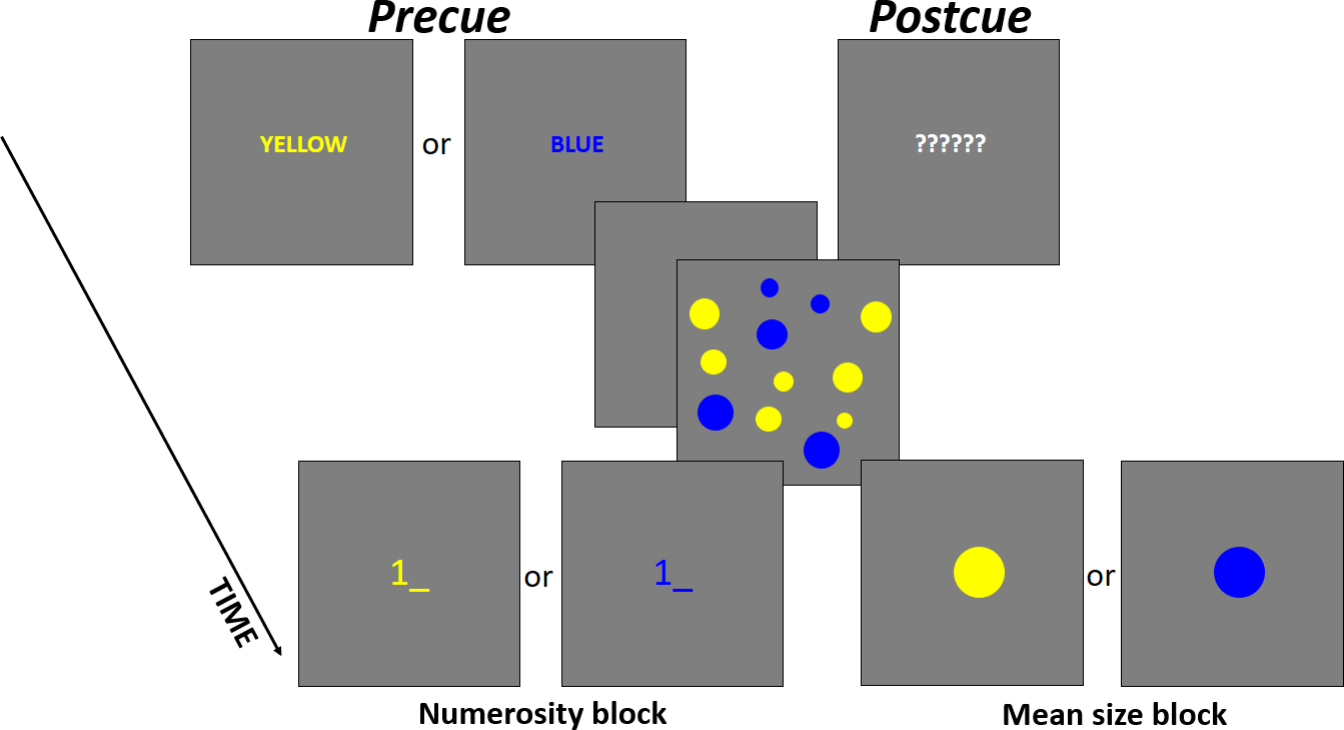
An example of a single trial with time course in Experiment 2. (A) precue and (B) postcue conditions.

Apparatus and stimulation were the same as in Experiment 1, except for a few important differences. Circles in a whole sample set (“the superset”) were colored in yellow or blue and intermixed by color in the space of a screen (Fig 3). This led the superset to form two overlapping color subsets. The total number of circles in a set varied from 10 to 36. As can be noted, the lower limit of the total number was higher than in Experiment 1. This was done to ensure that the number of circles within any subset was no fewer than five. We did not use subsets containing under five circles because small sets of objects (1 to ~4) are typically enumerated differently than larger sets [3]. The maximum number of circles in a subset was 23.

The mean size and standard deviation of the circles of the supersets ranged exactly as in Experiment 1. The mean size of each subset differed, on average, by 21% from the mean size of the other subset in a display and by 11% from the overall mean size of all circles. These differences ensured that participants could not reliably report the mean size of a given subset by deriving it from another attended subset or from the entire set. This encouraged the participants to divide their attention between the subsets when it was demanded by the task. As the standard deviations of the supersets were inherited from Experiment 1, keeping the systematic differences between mean sizes in the subsets inevitably reduced the local standard deviations in the subsets. They ranged from .05° to .19 (.10° on average).

For the precue, the Russian equivalents of either the word “YELLOW” (printed with a yellow font) or “BLUE” (printed with a blue font) were used. For the postcue, six question marks were presented, printed with a neutral white font. The cursor for typing a number and probe circles for adjusting the mean size could also be yellow or blue.

#### Procedure

As in Experiment 1, the participants had to report either the number or the mean size of circles presented in a sample set. The time course of a trial was the same as in Experiment 1. However, there were some important differences. Instead of reporting the relevant summary of the whole set, the participants were instructed to report the summary of a yellow or a blue subset. “Number” trials were presented in a separate block from “mean” trials, so a relevant parameter was always known to the participants. Therefore, their attention was always focused on one summary but could be divided between two subsets.

In the precue condition, a word “YELLOW” or “BLUE” appeared at the beginning of the trial informing the participant about the color of a relevant subset (Fig 3). In the postcue condition, six question marks replaced the cue. 500 ms after the cue, a sample set was presented for 500 ms, followed by the cursor or a probe circle (Fig 3). The cursor or a circle had the color of the relevant subset. That informed the participant about the relevant subset in the postcue trials and served as a reminder in the precue trials.

It is important to note that, in trials where one (the smaller) of the subsets contained fewer than five items, reports were demanded only on a larger subset, both in the numerosity and the mean size task. Nonetheless, the participants were not warned that they would never be asked about the small sets, so they divided their attention between both subsets anyway (unless the relevant subset was precued).

#### Design and data analysis

In this experiment, we used a 2 (Cue–precue vs. postcue) × 2 (Task–numerosity vs. mean size estimation) within-subject design. The number of trials per condition was 54 (two repetitions of 27 displays covering the whole range of numbers from 10 to 36). The experiment was divided into two blocks, one for the numerosity task and another for the mean size task. The order of block presentation varied across participants. The total number of trials in the two blocks was, therefore, 2 × 2 × 54 = 216 trials presented in a random order within each block (108 trials per block). Each block was preceded by 8 practice trials that were not included in the data analysis.

Data analyses were the same as in Experiment 1.

### Results and discussion

**Fig 4:**
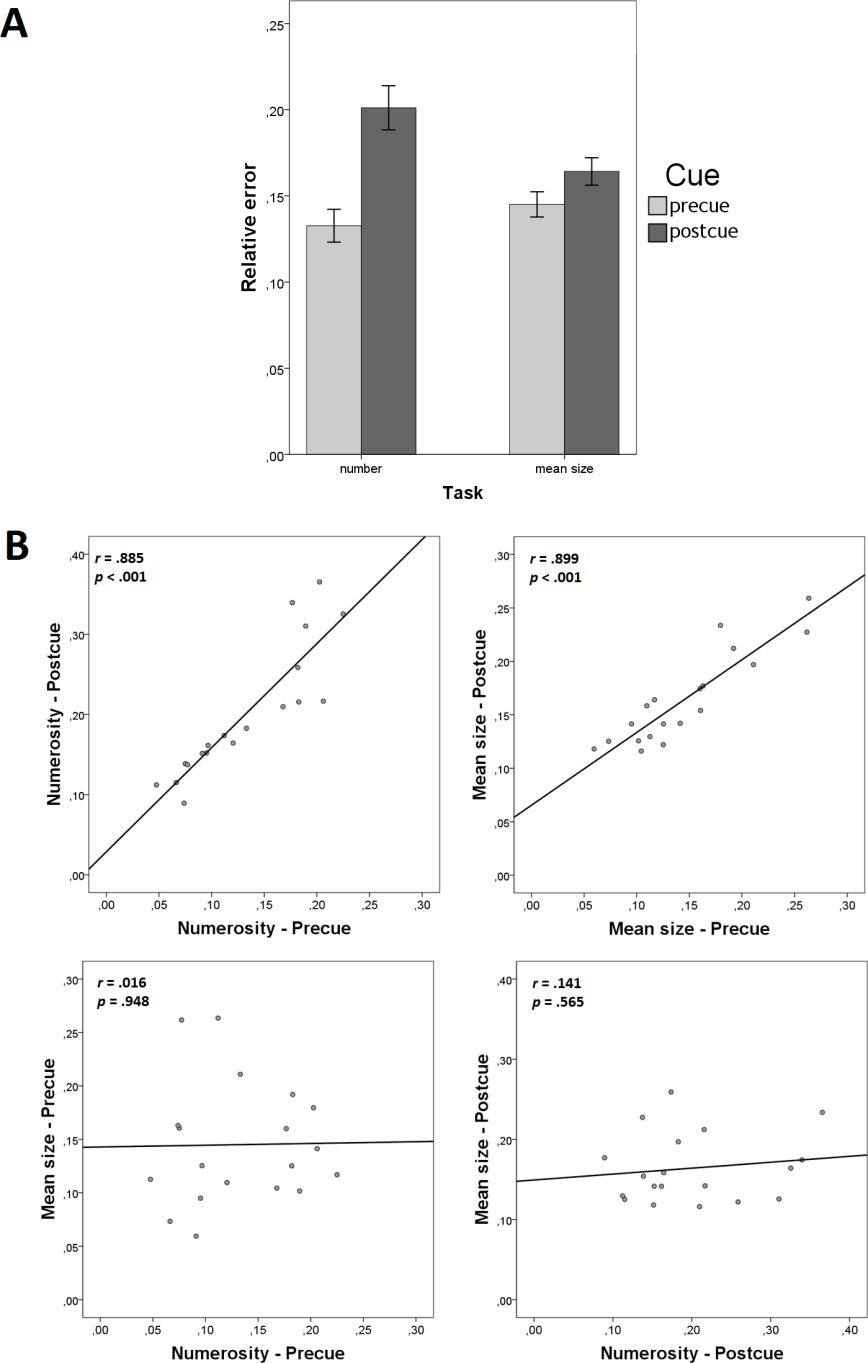
Results of Experiment 2. (A) cue effects on the relative error of numerosity and mean size judgments (error bars denote ±95% CI); and scatterplots showing (B) precue-postcue correlations within summaries and (C) between-summary correlations within different cue conditions.

We found a strong effect of Cue on the relative error (Fig 4A) in the numerosity task (*t*(18) = 7.273, *p* < .001, *d* = 1.669, *BF*_10_ = 19202.171) and a moderate effect in the mean size task (*t*(18) = 3.196, *p* = .005, *d* = .733, *BF*_10_ = 9.287). In both cases, the precue led to smaller relative errors compared to the postcue (Fig 4A). We therefore found evidence for the cost of dividing attention between two summaries of the same type when they belong to different sets.

Fig 4B and 4C illustrate the informative correlations. The results were very similar to the results of Experiment 1. Precue-postcue correlations were high within each of the summaries (“Numerosity—Precue” with “Numerosity—Postcue”: *r* = .885, *p* < .001, *BF*_10_ = 73149; “Mean size—Precue” with “Mean size—Postcue”: *r* = .899, *p* < .001, *BF*_10_ = 78566; Fig 4B) showing high consistency between the measurements across the cue conditions. We also found no evidence for correlations between different summaries within same-cue conditions (“Numerosity—Precue” with “Mean size—Precue”: *r* = .016, *p* = .938, *BF*_10_ = .284; “Numerosity—Postcue” with “Mean size—Postcue”: *r* = .141, *p* = .565, *BF*_10_ = .339; Fig 4C). These results replicated the absence of correlation that supports the validity of the conclusion from Experiment 1, that there is probably no common mechanism in the processing of numerosity and the mean.

In Experiment 2, the participants did not divide their attention between numerosity and the mean, as each of the tasks was presented as a separate block. Instead, the participants divided their attention between two numerosities or two mean sizes of different subsets. We found a cost of dividing attention under this condition compared to focusing on only one summary. These results somewhat contradict previous work showing that observers are equally good at representing the numerosities [5] and mean sizes ([54], Experiment 3; see also [28]) of two subsets under both the precue and postcue conditions. One difference that could explain the discrepancy between our data and that of Chong and Treisman [54] was the sensitivity of the test. While Chong and Treisman [54] used a two-alternative choice (their participants decided which of two probes had the mean size), our participants performed continuous adjustment, increasing or decreasing the probe in a pixel-by-pixel manner. These continuous estimates could be sufficient to reveal slight but significant differences between the precue and postcue conditions. However, this speculation requires further experiments to be tested. Halberda et al. [5] used a very similar procedure for number estimation to ours and even more difficult presentation conditions (their samples were masked both forward and backward) and found that most of their participants had shown no postcue cost for up to two subsets. However, Poltoratski and Xu ([59], Experiment 1), in their replication of Halberda et al. [5]’s study, found evidence for that cost for two subsets. It appears, therefore, that some differences in stimuli, procedures, or samples of observers can have something to do with the discrepancies in the results. More importantly, most of the mentioned studies explicitly show that the concurrent computation of summary statistics in different sets is prone to severe capacity limitations [28,30,31], which can be probed with the precue [5,59]. It also appears that, in Experiment 2, we found those limitations for both numerosity and the mean when they are tested independently. This finding is a baseline for Experiment 3, where the two summaries are estimated in different subsets concurrently.

## Experiment 3

### Method

#### Participants

Twenty-three psychology students of the Higher School of Economics took part in the experiment for extra course credit (18 females, ages ranging between 18 and 22 years). All reported having normal or corrected-to-normal vision and no neurological problems. The participants were unaware of the purpose of the experiment. Before beginning the experiment, they signed an informed consent for the procedure approved by the Institutional Review Board at HSE.

#### Apparatus and stimuli

Apparatus was the same as in the previous experiments. Stimulation was the same as in Experiment 2 concerning sample sets and report screens. Cue screens were the same as in Experiment 1 (words “NUMBER” and “MEAN” as precues, or a string of question marks as postcues) with an addition that the precue words could be colored in blue or yellow.

#### Procedure

**Fig 5:**
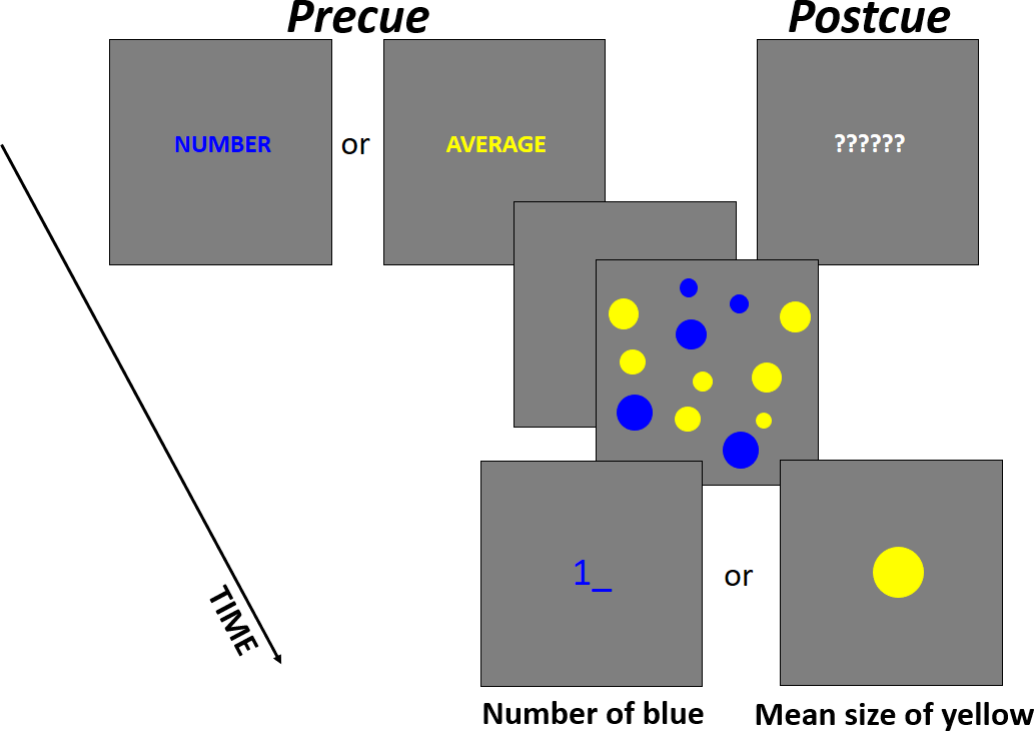
An example of a single trial with time course in Experiment 3. (A) precue and (B) postcue conditions.

In Experiment 3, participants were instructed to report the number of one color subset and the mean size of another one. For example, if a relevant subset on a trial was yellow, then the participant had to report its numerosity; if a relevant subset was blue, then the participant had to report its mean size (Fig 5). The participants had, therefore, to encode two different summaries–one from each subset [55]. In precue trials, a word informing about the relevant summary was presented on the cue screen. The precue word was printed in a color corresponding to the color of a subset assigned to the requested summary (Fig 5). In postcue trials, six white question marks appeared on the cue screen. As in the previous experiments, either the cursor or a probe circle appeared at the end of trial. These were also colored in accordance with the relevant subset (Fig 5). The rest details of the procedure were same as in previous experiments.

#### Design and data analysis

The design and the number of trials per condition were the same as in Experiment 2. There were only two differences. First, numerosity and mean size trials were randomly intermixed rather than blocked. Second, the colors of “numerosity” and “mean” trials varied across participants: One half of the participants had to report the number of yellow and the mean size of blue circles, while another half had to report the number of blue and the mean size of yellow circles. Only one practice block of 8 trials preceded the experimental block.

Data analyses were the same as in the previous two experiments.

### Results and discussion

The data of one participant were removed from the analysis as an outlier because of the systematic tendency to overestimate the number of circles by approximately 100% on average. This tendency led to the relative error in the numerosity task far exceeding three standard deviations. Therefore, data from 22 participants were used for further analysis.

**Fig 6:**
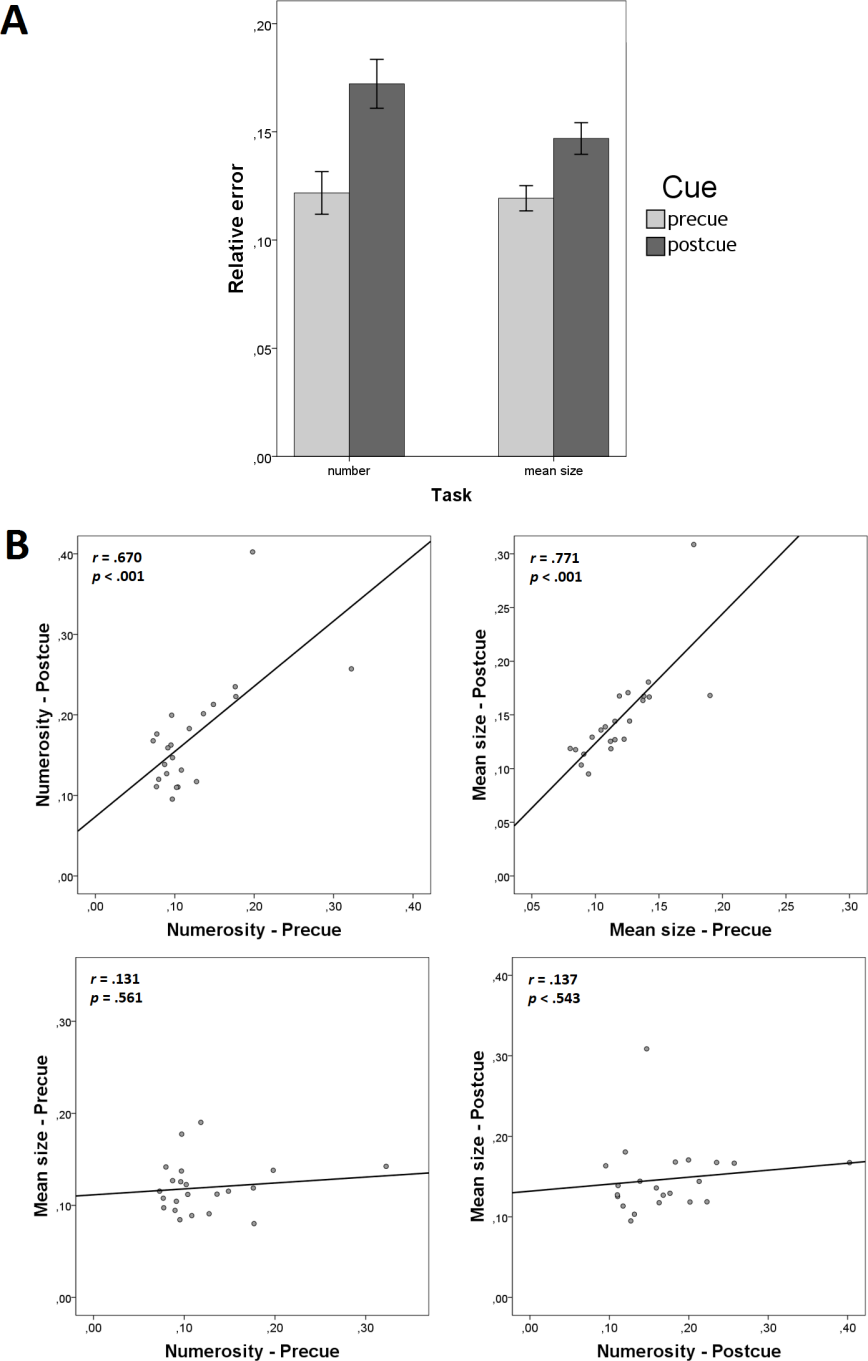
Results of Experiment 3. (A) cue effects on the relative error of numerosity and mean size judgments (error bars denote ±95% CI); and scatterplots showing (B) precue-postcue correlations within summaries and (C) between-summary correlations within different cue conditions.

We found strong effects of Cue on the relative error (Fig 4A), both in the numerosity task (*t*(21) = 4.337, *p* < .001, *d* = .925, *BF*_10_ = 106.3) and in the mean size task (*t*(21) = 4.597, *p* < .001, *d* = .980, *BF*_10_ = 184.4). In both cases, the precue led to smaller relative errors compared to the postcue (Fig 6A). These results suggest that there was a cost of dividing attention between numerosity and the mean size when these summaries belong to different sets.

Fig 6B and 6C illustrate the informative correlations. Precue-postcue correlations were high within each of the summaries (“Numerosity—Precue” with “Numerosity—Postcue”: *r* = .670, *p* = .001, *BF*_10_ = 48.163; “Mean size—Precue” with “Mean size—Postcue”: *r* = .771, *p* < .001, *BF*_10_ = 953.552; Fig 6B) showing high consistency between the measurements across cue conditions. Correlations between different summaries within same cue conditions were non-significant (“Numerosity—Precue” with “Mean size—Precue”: *r* = .131, *p* = .561, *BF*_10_ = .320; “Numerosity—Postcue” with “Mean size—Postcue”: *r* = .137, *p* = .543, *BF*_10_ = .315; Fig 6C). This pattern replicates the patterns from Experiments 1 and 2, suggesting the absence of a common mechanism in the processing of numerosity and the mean. We conclude that these two summaries are processed independently.

Like in Experiment 1, our participants had to divide their attention between numerosity and the mean size on postcue trials. Like in Experiment 2, they also divided their attention between subsets when estimating those statistics. We found evidence for some cost of divided attention that we did not observe in Experiment 1. However, a similar cost was found in Experiment 2 when only one of the “processors” was always enabled on each block of trials. Therefore, we can conclude that the postcue cost in Experiment 3 was likely to be caused by dividing attention between two subsets rather than two summary statistics.

## General discussion

Summarizing the results from Experiments 1–3, we came to three important findings. First, our observers had no difficulties in dividing their attention between the numerosity and the mean size of the same set (Experiment 1). Second, there was no correlation between the precision of numerosity and mean judgments, and this result was highly consistent across all experiments and conditions. Third, the division of attention between same-type (Experiment 2) or different-type (Experiment 3) summaries yielded some costs if these summaries belonged to different sets. We discuss the theoretical implications of these findings below.

### Independence and parallelism: Implications for visual statistical processing

In our experiments, we combined a correlational approach [48,60] with the precue-postcue paradigm [5,54,55,57] in order to test correlation and parallelism between two domains of ensemble summary statistics–numerosity and mean size. As we noted above, both these tests focus on different aspects of the functional organization of ensemble perception. Without testing parallelism, the observed uncorrelatedness (independence) only shows the absence of a potential overlap between different domains, but not their coordination in conscious perception when multiple statistics are required for a rich description of a stimulus. Likewise, without testing correlation, it is hard to say whether the observed parallel statistical computations are provided by truly independent domains or by a unitary algorithm that comprises all computations (e.g., computing the average using numerosity information). Experiment 1 was the main and most straightforward test for both correlation and parallelism, given that only one ensemble has always been processed but two different statistical summaries have been extracted. The results of this experiment showed consistency with an idea of two well-separated domains for numerosity and the mean size, as we found no correlation between these tasks and no signs of interference.

In Experiments 2 and 3, we tested the tasks in which the visual system was busy with two summaries of different types, with the tasks in which it was busy with two same-type summaries. This testing scheme allowed us to compare the supposed distributed computation (when coordination between the domains is required) with shared computation (when no coordination is required because only one processor is always used). We also addressed the controversial issue of dividing attention between summaries vs. sets. It turned out that both distributed and shared computations showed the cost of divided attention. The whole triplet of the experiments eventually provided us with important information about the capacity limitations of ensemble summary statistics. Comparing Experiments 1 and 3, both requiring distributed computation but differing in the number of attended sets, we can see that it is attending to two sets that causes interference. Therefore, when we turn to Experiment 2, requiring the shared computation of two same-type statistics in two sets, we should conclude that there are probably no specific constraints in computational capacities per se, even when they share a single mechanism. This conclusion is supported by the high precue-postcue correlations in same-type summaries in Experiment 2: These correlations show that, if ensemble summary is estimated precisely in one ensemble, it is also estimated precisely in two ensembles, yet with some loss in both. In line with other previous studies [57–59], we conclude that capacity limitations of ensemble summary statistics are associated with the limits in parallel encoding of multiple sets. Beyond this limitation, computational capacity for estimating summary statistics can be rather large, both in distributed and shared computations.

An idea of a single “statistical processor” performing numerous ensemble computations looks intuitively appealing when we borrow terms and concepts from a different discipline–regular mathematical statistics–to describe a part of visual reality. In addition, it seems even more plausible given interesting data in favor of a possible connection between some ensemble statistics (numerosity) and more generic and not purely visual mathematical abilities [67]. However, our results, along with the previously reported data [48,60], go against the idea of such a general processor. Perhaps some overlap (correlation or interference) between ensemble summaries is possible, but so far it has been documented only between a few combinations of basic features–mean size and mean speed, mean size and mean orientation [55], mean orientation and mean color [48]. For more “remote” summaries, such as numerosity and the mean size, there is no evidence for such an overlap.

The parallel and independent character of numerosity and mean size processing leads us to an idea of a more modular (domain-specific) architecture [68] underlying a variety of phenomena referred to as ensemble summary statistics. Modularity implies that a number of highly domain-specific structures work on various ensemble properties, each within its domain specificity, and the modules do not overlap. Our claim that the computation of numerosity and the mean size are provided by separate domain-specific “modules” is supported by neural data. It is shown that numerical functions, including numerosity estimation in multiple objects, are associated with increased activity in the parietal cortex, especially the intraparietal sulcus [69,70], and activity in the prefrontal cortex [70]. On the other hand, Cant and Xu [23] have recently shown that the processing of shape statistics in photorealistic multiple objects is associated with increased activity in the parahippocampal place area and lateral occipital area, known to respond to intrinsic properties of objects and scenes.

### How can ensemble summary statistics be calculated?

The second important issue associated with the links between the mean and numerosity is the question whether, like in regular statistics, calculating the mean requires information about the number (as the number of items in a sample is the denominator in the formula to calculating the mean). Applying this idea to our tasks, it could be supposed that, for size averaging, the visual system roughly estimates individual sizes and the number of circles, then sums the sizes, and divides by the number. However, the data from all three of our experiments show that this scenario is unlikely. If two processors, as shown, are independent, then they hardly “know” what calculations the other is doing and what result is being reached. This concept is supported by the absence of significant correlations between the precision of number estimation and that of mean size estimation, which is consistent with the conclusion made by Lee et al. [60].

The finding that the visual system does not act like a regular statistician while calculating the average makes us consider alternative mechanisms that could lie within a core of representing ensemble summary statistics. One approach that could suggest a potential explanation is based on the idea of *sampling*, which resonates particularly in studies of size and color averaging [37,38,40,41]. Sampling implies that the mean can be efficiently estimated based on just few items (2 to 4) that occur to be attended to and loaded into working memory. Therefore, if only a sample of items is averaged, then there is no need for the knowledge of the total number of items in a display: The system averages these few items and approximates the estimate for the rest, regardless of their number. However, a number of studies tend to disprove the limited-capacity sampling mechanism of averaging [30–34,36,71]. This also questions the sample-based explanation of the absence of links between the mean size and numerosity.

The second possible idea that potentially can explain how visual averaging bypasses operating numerosity is based on the concept of *pooling*. It refers to the mechanism of accumulating signals by higher levels of the visual hierarchy from its lower levels in the feedforward processing stream. The idea of pooling as a mechanism of representing summary statistics is widespread [10,25,43,72–74], and for ensemble averaging, it is well explained by Haberman and Whitney [42]. At the lower levels of analysis (for example, at the level of V1), every ensemble element is represented by a population response of feature-selective neurons with narrow receptive fields, that is, every such population is very locally tuned to a small piece of the visual field. On higher levels, receptive fields become larger, accumulating information from several lower receptive fields. Therefore, neurons in those large receptive fields can be selectively activated by a combination of lower-level signals. Presumably, the peak activation will be produced by neurons responsive to the average value of the lower-level signals [24]. That process can explain how the mean ensemble feature can be encoded directly as a sensory property [20]–which is of particular importance for our topic–without taking the number of elements into account. Nevertheless, our data reported in this article do not allow us to directly conclude which of the strategies is actually used for “numerosity-free” computation of the mean size. Further experiments are required to clarify this point.

## Conclusions

Numerous demonstrations of rapidly computed ensemble summary statistics raise the question of what performs these computations. Is it a single general structure that can perform a broad range of statistical transformations on data from different sensory domains? Our findings described in this article suggest that different statistical estimates–numerosity and the mean size–extracted from exactly the same stimulus are likely to be provided by independent domain-specific mechanisms, rather than by a single domain-general one. This result also implies that, unlike mathematical statistics, visual averaging does not operate information about the number of individual objects which, in turn, questions whether ensemble perception is based on a set of transformations reproducing regular statistics literally.

## Supporting information

S1 File Full raw data of all participants from Experiments 1–3. (RAR)
